# Transarterial chemo-embolisation of hepatocellular carcinoma: impact of liver function and vascular invasion

**DOI:** 10.1038/bjc.2016.423

**Published:** 2017-01-26

**Authors:** Imam Waked, Sarah Berhane, Hidenori Toyoda, Stephen L Chan, Nicholas Stern, Daniel Palmer, Toshifumi Tada, Winnie Yeo, Frankie Mo, Dominik Bettinger, Martha M Kirstein, Mercedes Iñarrairaegui, Asmaa Gomaa, Arndt Vogel, Tim Meyer, Bruno Sangro, Paul Lai, Takashi Kumada, Philip J Johnson

**Affiliations:** 1 Department of Hepatology, National Liver Institute, Menoufeya University, Menoufia Governorate, Egypt; 2 Department of Molecular and Clinical Cancer Medicine, University of Liverpool, The Sherrington Building, Ashton Street, Liverpool L69 3GA, UK; 3 Department of Gastroenterology and Hepatology, Ogaki Municipal Hospital, 4-86 Minaminokawa-cho, Ogaki, Gifu 503-8052, Japan; 4 Department of Anatomical & Cellular Pathology, Chinese University of Hong Kong, Hong Kong Cancer Institute, Hong Kong, China; 5 Digestive Diseases Unit, Aintree University Hospitals NHS Foundation Trust, University Hospital Aintree, Liverpool, UK; 6 State Key Laboratory in Oncology in South China, Sir Y. K. Pao Centre for Cancer, Department of Clinical Oncology, Chinese University of Hong Kong, Hong Kong Cancer Institute, Hong Kong, China; 7 Department of Internal Medicine II, University Hospital Freiburg, Hugstetter Street 55, Freiburg D-79106, Germany; 8 Department of Gastroenterology, Hepatology and Endocrinology, Medical School Hannover, Carl Neuberg Street 1, Hannover 30625, Germany; 9 Liver Unit and HPB Oncology Area, Clinica Universidad de Navarra; and Centro de Investigacion Biomedica en Red de Enfermedades Hepaticas y Digestivas (CIBEREHD), Pamplona, Spain; 10 Department of Oncology, UCL Cancer Institute, University College London, London, UK; 11 Department of Surgery, Prince of Wales Hospital, Chinese University of Hong Kong, Hong Kong, China; 12 The Clatterbridge Cancer Centre NHS Foundation Trust, Clatterbridge Road, Bebington, Wirral CH63 4JY, UK

**Keywords:** hepatocellular carcinoma, transarterial chemo-embolisation, ALBI grade, HAP score, vascular invasion, Child-Pugh

## Abstract

**Background:**

Transarterial chemo-embolisation (TACE) is recommended for patients with BCLC intermediate stage hepatocellular carcinoma (stage B), particularly in patients with good underlying liver function and minimal symptoms. The hepatoma arterial embolisation prognostic (HAP) score combines measures of liver function and tumour-related factors to offer a simple prognostic scoring system. The Albumin-Bilirubin (ALBI) grade permits assessment of the impact of liver function on survival. We aimed to investigate these two models and vascular invasion (VI).

**Methods:**

In an international cohort of 3030 patients undergoing TACE, we examined the impact of liver function as assessed by the ALBI score, the HAP score and VI on survival.

**Results:**

Classification according to ALBI grade resulted in non-overlapping survival curves in the overall data set and all regional cohorts. The HAP score was also validated. Tumour number, aetiology and VI were identified as additional independent prognostic risk factors not currently included in the HAP score. Survival was particularly poor for patients with VI.

**Conclusions:**

The ALBI grade categorised patients receiving TACE into three clear prognostic groups, thereby emphasising the importance of underlying liver function in the outcome of TACE. The HAP score has been validated internationally and the serious adverse impact of VI is clearly shown.

International guidelines recommend Transarterial chemo-embolisation (TACE) for patients with hepatocellular carcinoma (HCC) at the BCLC intermediate stage (B) or for those patients in the early stage that are not candidates for percutaneous ablation, liver resection or transplantation (Bruix and Sherman, 2011). This recommendation was based upon two randomised trials and a subsequent systematic review (Llovet *et al*, 2002; Lo *et al*, 2002; Llovet and Bruix, 2003). However, the benefits from TACE remain controversial (Marelli *et al*, 2007; Curley *et al*, 2012; Meyer *et al*, 2013). A recent Cochrane review concluded that there was no firm evidence to support or refute the benefit of TACE for patients with unresectable HCC (Oliveri *et al*, 2011), although this conclusion has, itself, been robustly challenged (Forner *et al*, 2012).

Nonetheless, there is little argument that the degree of underlying liver (dys)function is an important factor determining survival and hence in defining the patient groups most likely to benefit from this form of treatment. International guidelines suggest that TACE should be confined to those with Child-Pugh (C-P) ‘A' disease and that patients with vascular invasion (VI) should receive sorafenib rather than TACE although VI was not excluded in one of the original RCTs (Lo *et al*, 2002). However, it is now well-established that TACE can be performed safely in the presence of some degree of VI and, in current clinical practice, a significant proportion of patients undergoing TACE do, in fact, have VI. We have previously developed and validated a simple model, the hepatoma arterial embolisation prognostic (HAP) score (Kadalayil *et al*, 2013), based on a cohort of UK patients, that permits assessment of prognosis after TACE. The model was built on the clinical parameters of bilirubin, albumin, tumour size and Albumin-Bilirubin (AFP), the former two presumably reflecting the impact of liver function and the latter two, the impact of tumour-related factors, on survival.

We have now assembled a comprehensive global data set that includes patient level data from >3000 patients undergoing TACE and undertaken a rigorous statistical analysis of the factors influencing survival. We placed particular emphasis on underlying liver function and the presence or absence of VI and, in the process thereof, we sought to validate the HAP score. Liver function was assessed by applying the recently developed ALBI score (Johnson *et al*, 2014), a simple objective and extensively validated (Chan *et al*, 2015; Hiraoka *et al*, 2015; Chan *et al*, 2016), approach.

## Patients and methods

The cohorts comprised patients undergoing TACE in four different regions namely Europe, Japan, China (Hong Kong) and Egypt (Table 1). The European cohort comprised 413 patients from the UK (Birmingham, Liverpool and London), 731 from Germany (Hannover and Freiburg) and 88 patients from Pamplona, Spain. Some of the data from the UK (London) came from those on which the HAP score was originally derived. These patients were excluded for the HAP score validation. The Japanese cohort (*n*=655) were recruited from five institutions in the Western part of Japan, as previously reported by Toyoda *et al* (2006). The Chinese patients (*n*=145) were from those attending the Joint Hepatoma Clinic at the Prince of Wales Hospital, Hong Kong and the Egyptian cohort (*n*=998) were from those referred to the Oncology department of the National Liver Institute in Shebeen ElKom, Egypt. Detailed demographic data is given in Table 1. Data recorded in each cohort include age, gender, albumin (g l^−1^), bilirubin (μmol l^−1^), tumour number (solitary or multiple), tumour size (cm), VI, AFP (ng ml^−1^), C-P grade and aetiology (HCV, HBV or ‘other'). ‘Other' comprised mainly patients with alcoholic liver disease. Laboratory data were recorded within the 6 week period before the first TACE procedure which was, in turn, undertaken within 6 weeks of diagnosis. Vascular invasion (including portal vein, hepatic vein and inferior vena cava involvement) was assessed in the portal phase of computed tomography and, supplemented where appropriate, by arterial portography. Assessments were made within the 6 week period before treatment. In the Japanese cohort detailed information concerning the extent of portal vein invasion, ranging from VP0 to VP4 was recorded. VP0 indicated no tumour thrombus in the portal vein; VP1, tumour thrombus distal to, but not involving, the second-order branches; VP2, tumour thrombus in the second-order branches; VP3, tumour thrombus in the first-order branches; and VP4, tumour thrombus in the main trunk (Katagiri and Yamamoto, 2014; Kudo *et al*, 2015). For the purposes of the present analysis, VP2 and VP3 were combined.

The centres involved had extensive experience in the management of HCC and the use of TACE. We included all patients that were classified by the local investigator as undergoing TACE as their primary treatment, excluding only those where TACE was used as a bridge to transplantation or other potentially curative treatment options. Neither the response, nor any specific aspects of the procedure such as type of cytotoxic drug or embolic agent used or frequency of repeat TACE, or other treatment after the primary treatment, were recorded for the purpose of this study. All data were analysed in the UK (University of Liverpool) and used exactly as presented by the contributing investigator. Liver function was assessed by the C-P grade (as graded by the local investigator) and the ALBI score, the latter being graded according to the published cutoff points. Grades 1, 2 and 3 refer to good, intermediate and poor liver function, respectively.

### Statistical methods

All statistical analyses were undertaken using Stata/SE 14.1 (StataCorp LLC, Lakeway Drive, TX, USA). The HAP score (Kadalayil *et al*, 2013) and ALBI grade (Johnson *et al*, 2014) were calculated as previously described. Survival (in months) was calculated from date of TACE treatment until date of death or date of last follow-up. Survival according to HAP score or ALBI grade was plotted using the Kaplan–Meier method. The different classification systems were compared using the Harrell's C (a measure of predictive power; Harrell *et al*, 1982; Harrell *et al*, 1996; Newson, 2010) and Akaike information criterion (AIC; a measure of model fit; Akaike, 1998). Higher values of the former and lower values of the latter indicates a better prognostic utility of the model. Log-rank tests were used to compare between the survival curves within each staging group. Alphafetoprotein (AFP) and bilirubin were log-transformed due to extreme skewness. Variables that influence survival were identified using univariable Cox proportional hazards model. Using forward selection of variables at the *P*=0.05 level (and likelihood ratio test at each step), a multivariable Cox proportional hazards model was built to explain survival in TACE patients. For the Cox regression models, all the cohorts were merged and ‘region' was used as a frailty term. The proportional hazards assumption was tested on the basis of scaled Schoenfeld residuals after fitting the Cox regression model. For all tests, statistical significance level was set at 5%.

## Results

**Figure 1:**
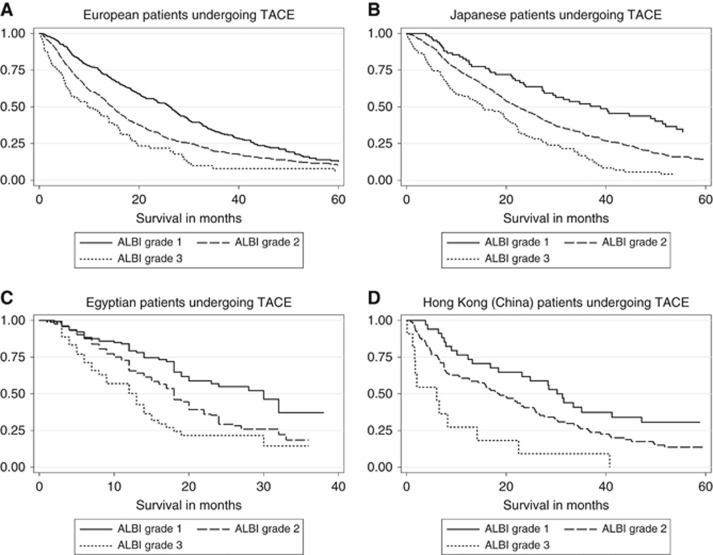
**Kaplan–Meier curves depicting survival according to ALBI grade.** Patients are classified as undergoing TACE from (**A**) Europe, (**B**) Japan, (**C**) Egypt and (**D**) Hong Kong, China.

**Figure 2:**
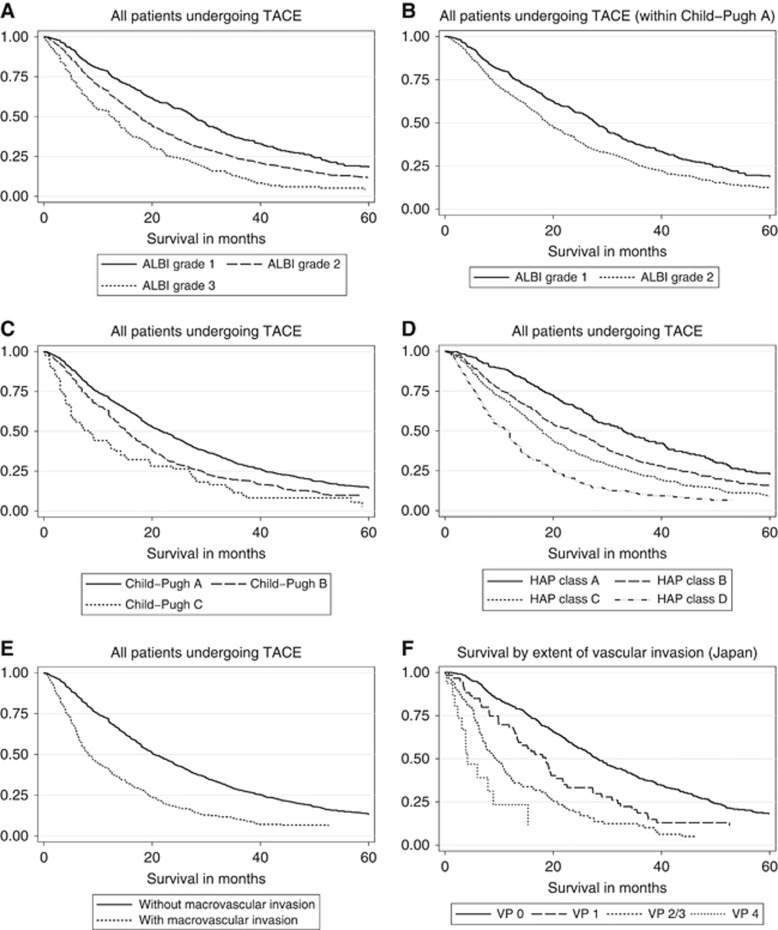
**Kaplan–Meier curves depicting survival in all the patients undergoing TACE.** Patients are classified according to (**A**) ALBI grade, (**B**) ALBI grade within C-P grade A, (**C**) C-P grade, (**D**) HAP class, (**E**) the presence or absence of macrovascular invasion and (**F**) the extent of vascular invasion (Japan).

The results from the univariable Cox regression analysis (Table 2) showed that albumin, bilirubin, tumour number, tumour size, VI and aetiology were prognostic in patients undergoing TACE (all *P*⩽0.0001). The multivariable Cox regression model (Table 3) showed that the key variables influencing survival were related to tumour characteristics (tumour size, tumour number, AFP and VI; *P*<0.0001), liver function (albumin and bilirubin) (*P*<0.0001), aetiology (*P*=0.0082) and age (*P*=0.0012; Table 3). Effect of age on survival was more notable in those over 70 years of age compared with the other age groups (Table 3; Supplementary Table 1). Most patients had CP grade ‘A' liver function; the remainder had CP grade B with only a small percentage (4%) having CP grade C (Table 1). Assessing liver function by the two variables of bilirubin and albumin as defined in the ALBI score revealed a clear discrimination in survival between each ALBI grade in all separate regions (Figures 1A–D) and overall (Figure 2A). The model also revealed clear separation within C-P ‘A' patients (Figure 2B; Supplementary Figures 1a–d). Assessing liver function with the C-P score also showed separation by grade in each individual region (Supplementary Figures 2a–d). There was no clear difference between C-P B and C-P C particularly amongst the European, Japanese and Hong Kong cohorts but, as expected, the numbers in the C-P C group were low. Results after merging the data sets from all four regions showed clear separation between the three C-P grades (Figure 2C). Both by visual inspection and formal statistical analysis (via Harrell's C statistic), the ALBI score is at least as effective as C-P in discriminating between prognostic groups. Harrell's C statistic was 0.5661 and 0.5586 for ALBI and C-P, respectively. The corresponding AIC values were 26963.33 and 26548.21, respectively.

The HAP score was originally developed using the UK data sets only (London). Applying the HAP score to each of the other cohorts – Europe (Liverpool, Birmingham, Spain and Germany), Japan, Egypt and Hong Kong – produced four prognostic groups in each region (Supplementary Figures 3a–d) and overall (Figure 2D), thereby extending the generalisability of the score, although it should be noted that there was considerable overlap between HAP 2 and 3 in the Egyptian (Supplementary Figure 3c), and to a lesser extent the Hong Kong cohorts (Supplementary Figure 3d). Merging all the cohorts, however, generated four clear prognotic groups (log-rank tests, *P*<0.0001 for all combinations; Figure 2D).

Overall 15% of patients had VI and there was a very clear difference in survival according to presence or absence thereof, in all regions (Supplementary Figures 4a–d) and overall (Figure 2E). Among those with VI survival was particularly poor, ranging from 2.7–10.7 months in the various regions and 8.2 months overall (Supplementary Figures 4a–d; Figure 2E). In the Japanese cohort, there was clear deterioration in overall survival with increasing extent of the portal vein involvement (Figure 2F).

Median survival (and 95% CI) for each of the above sub-groups, as well as the Harrell's C and AIC scores are summarised in Table 4 and Supplementary Table 2. Log-rank test outcomes for each combination of the survival curves in all the figures is summarised in Supplementary Table 3.

## Discussion

Our multivariable analysis showed that the key variables influencing survival were related to tumour characteristics (tumour size, tumour number, AFP and VI), liver function (albumin and bilirubin) and aetiology. These results were largely in agreement with the literature (Savastano *et al*, 1999; Lladó *et al*, 2000; O'Suilleabhain *et al*, 2003; Grieco *et al*, 2005; Takayasu *et al*, 2006) and our previous analysis (based largely on the current UK data set). In the latter, we used bilirubin, albumin, tumour size and AFP to develop a score (the HAP score) that gave accurate and nationally validated prognostication (Kadalayil *et al*, 2013). The present study lends international support to the HAP score, but as noted above there is considerable overlap between HAP stages B and C in the Chinese and Egyptian cohorts.

However, the data set on which the HAP score was originally developed did not contain information on tumour number and the number of patients with different aetiologies and VI was too small for meaningful statistical analysis; these parameters did not, therefore, enter the model. Thus, it became apparent in the present, much larger and international data set, that VI, tumour number, age and aetiology were also important, and these factors could be added to the HAP score to increase its prognostic utility. It would be possible to build a more rigorous model involving these variables, but the inevitable increase in complexity would detract from relative simplicity and ready clinical applicability of the currently formulated HAP score. Our data sets were accrued before the recent publication of other scoring systems designed to facilitate identification of patients appropriate for TACE and we have not therefore collected the variables that would be required for comparison with HAP (Hucke *et al*, 2014; Adhoute *et al*, 2015).

Our results also give particular insight into two of the key prognostic variables that are integral to the model namely liver function and VI. Thus albumin and bilirubin are clear measures of liver (dys) function and form the basis of our ALBI score. In all regions there is clear discrimination in survival according to ALBI and this was also maintained within patients classified as C-P ‘A'. Despite this clear discrimination within regional groups, the percentage falling into each ALBI group and the survival within each group was different. This presumably reflects the different availability of, and indication for, various therapeutic options and treatment algorithms in the different regions and the different aetiologies. For example, Sorafenib was not available in Egypt and liver transplantation was not available in the Japanese or Hong Kong cohorts. It should be noted here that the ALBI score is not an HCC staging system since it only measures liver function, taking no account of tumour-related factors. To this extent it is not directly comparable to the HAP score which was derived to assess survival after arterial therapies by combining both liver function and tumour-related factors.

Our current analysis suggests that the ALBI score is at least as discriminatory, in prognostic terms, as the CPS in the TACE setting and in a recent study on a similar population, but including those undergoing radio-embolisation, Hickey *et al* (2016) concluded that the ALBI score ‘outperformed' the CPS in discriminating survival. Even without any claim to superiority over the CPS, the ALBI score/grade has several advantages. Specifically, it does not require three of the five parameters involved in the CPS (including the two that are most subjective, ascites and encephalopathy), thereby making the classification more reliable between observers. A less obvious advantage is that it was built on an extensive evidence base, specifically for assessment of liver function in HCC and made no prior assumptions as to the presence or absence of cirrhosis. By contrast, the CPS is advocated for the assessment of liver function in patients with cirrhosis and, at least by convention, it should only be applied to such patients. This convention is generally not applied to patients with HCC being used widely, even with the knowledge that many patients with HBV-related HCC will not have cirrhosis. In our recent study, where we examined the extent of fibroses in resected HCC specimens and even among the Japanese patients, most of whom had HCV-related HCC, only 52% had cirrhosis (Toyoda *et al*, 2016a). It should not be assumed therefore that, because all patients with HCC were assigned a CPS by their local investigator, that all had cirrhosis.

The prevalence of VI (15%) is in line with reports in the literature (Pirisi *et al*, 1998; Minagawa and Makuuchi, 2006; Yoshidome *et al*, 2011; Quirk *et al*, 2015; 10–40%) and confirms that despite guidelines, suggesting that patients with VI should receive sorafenib, a significant proportion of those undergoing TACE did in fact have VI. The prognostic importance of VI is also seen in patients treated with curative intent (Toyoda *et al*, 2016b). Although the AASLD/EASL guidelines (Llovet *et al*, 2008a) suggest the treatment with sorafenib rather than TACE for those with any degree of VI there is no explicit statement that suggests that VI is a contraindication to TACE and the data presented here suggest the extent of portal vein invasion is of major prognostic importance. Quirk *et al* (2015), have noted that overall survival after TACE in patients with VI ranges from 7.4 to 10.2 months in the literature (and in the series reported here), figures that are only marginally better than those obtained with systemic sorafenib (Llovet *et al*, 2008b).

## Figures and Tables

**Table 1:** Demographics

**Variables**	**Europe (*****n*****=1232)**	**Japan (*****n*****=655)**	**Egypt (*****n*****=998)**	**Hong Kong (*****n*****=145)**	**All (*****n*****=3030)**
% Male	83.12, *n*=1232	74.66, *n*=655	83.27, *n*=998	85.52, *n*=145	81.45, *n*=2769
Age (median, IQR)	66.48 (59.00–73.18), *n*=1230	65.00 (58.00–73.00), *n*=655	57.00 (51.00–62.00), *n*=998	65.00 (56.00–71.00), *n*=145	62.77 (55.00–70.07), *n*=3028
Albumin, g l^−1^ (median, IQR)	37.00 (33.00–41.00), *n*=1214	32.00 (28.00–36.00), *n*=653	33.00 (29.00–37.00), *n*=998	35.00 (32.00–39.00), *n*=145	35.00 (30.00–39.00), *n*=3010
Bilirubin, μmol l^−1^ (median, IQR)	16.00 (10.94–26.00), *n*=1213	15.39 (11.97–23.94), *n*=653	22.23 (13.68–32.49), *n*=998	14.00 (9.00–22.00), *n*=145	17.10 (11.97–27.36), *n*=3009
Tumour size, cm (median, IQR)	5.00 (3.40–7.60), *n*=1180	3.50 (2.20–5.40), *n*=594	6.00 (4.00–8.00), *n*=998	6.00 (3.80–10.00), *n*=141	5.00 (3.40–7.60), *n*=2913
% Multifocal	66.24, *n*=1161	69.27, *n*=654	75.75, *n*=998	58.33, *n*=144	69.73, *n*=2957
AFP, ng ml^−1^ (median, IQR)	46.00 (6.70–534.50), *n*=1045	45.45 (12.40–510.00), *n*=614	129.00 (17.00–600.00), *n*=998	92.00 (10.00–1365.00), *n*=145	70.45 (11.00–584.00), *n*=2802
INR (median, IQR)	1.10 (1.01–1.20), *n*=851	NA	1.40 (1.20–1.70), *n*=998	1.14 (1.07–1.22), *n*=145	1.20 (1.10–1.40), *n*=1994
ALBI score (median, IQR)	−2.34 (−2.72 to −1.91), *n*=1203	−1.97 (−2.36 to −1.55), *n*=653	−1.91 (−2.34 to −1.55), *n*=998	−2.29 (−2.59 to −1.90), *n*=145	−2.10 (−2.53 to −1.67), *n*=2999
% ALBI grade (1 : 2 : 3)	32.09 : 60.93 : 6.98, *n*=1203	13.02 : 70.75 : 16.23, *n*=653	15.63 : 69.14 : 15.23, *n*=998	24.83 : 67.59 : 7.59, *n*=145	22.11 : 66.12 : 11.77, *n*=2999
% Child-Pugh (A : B : C)	73.94 : 24.62 : 1.45, *n*=1174	52.06 : 41.22 : 6.72, *n*=655	47.60 : 46.89 : 5.51, *n*=998	77.24 : 21.38 : 1.38, *n*=145	60.43 : 35.60 : 3.97, *n*=2972
% HAP class (A : B: C: D)	18.72 : 34.42 : 31.95 : 14.91, *n*=892 (Liverpool, Birmingham, Germany and Spain only)	13.49 : 32.40 : 38.53 : 15.59, *n*=571	9.02 : 22.55 : 41.08 : 27.35, *n*=998	12.06 : 37.59 : 29.79 : 20.57, *n*=141	13.49 : 29.59 : 36.78 : 20.14, *n*=2602
% Vascular invasion	10.61, *n*=1225	30.28, *n*=654	10.32, *n*=998	11.03, *n*=145	14.79, *n*=3022
% HCV : HBV : other	24.29 : 12.19 : 63.52, *n*=1165	56.66 : 18.42 : 24.92, *n*=646	98.39 : 1.10 : 0.50, *n*=996	8.33 : 79.86 : 11.81, *n*=144	55.61 : 13.11 : 31.28, *n*=2951
Overall survival (months, 95% CI)	16.6 (15.4–18.0), *n*=1226	22.3 (20–24.5), *n*=655	18.0 (17.0–19.0), *n*=998	19.9 (14.2–25.6), *n*=143	18.6 (17.9–19.5), *n*=3022

Abbreviations: AFP=alphafetoprotein; ALBI=Albumin-Bilirubin; CI=confidence interval.

**Table 2:** Univariable Cox regression

**Variable**	**Hazard ratio (95% CI)**	***P*****-value**	**LR test of theta=0**
Age groups			
Under 51	1		
51–60	0.967 (0.822–1.138)	0.688	
61–70	0.914 (0.779–1.072)	0.269	
Over 70	0.950 (0.805–1.122)	0.546	0.003
Gender			
Female	1		
Male	1.099 (0.980–1.234)	0.108	0.005
Albumin (g l^−1^)	0.958 (0.951–0.966)	<0.0001	<0.0001
Log 10 bilirubin	1.894 (1.608–2.231)	<0.0001	0.007
Tumour number			
Solitary	1		
Multiple	1.279 (1.158–1.412)	<0.0001	0.005
Tumour size (cm)	1.072 (1.059–1.086)	<0.0001	0.084
Vascular invasion			
No	1		
Yes	2.282 (2.024–2.573)	<0.0001	<0.0001
Log 10 AFP	1.263 (1.215–1.312)	<0.0001	<0.0001
Aetiology			
HCV	1		
HBV	1.211 (1.055–1.391)	0.007	
Other	1.276 (1.138–1.432)	<0.0001	0.077

Abbreviations: AFP=alphafetoprotein; CI=confidence interval; HCV=hepatitis C virus; HBV=hepatitis B virus.

**Table 3:** Multivariable Cox regression

**Variable**	**Hazard ratio (95% CI)**	***P*****-value**
Vascular invasion		
No	1	
Yes	1.751 (1.515–2.025)	<0.0001
Albumin (g l^−1^)	0.959 (0.950–0.968)	<0.0001
Log10 AFP	1.209 (1.158–1.261)	<0.0001
Tumour size (cm)	1.054 (1.038–1.070)	<0.0001
Log10 bilirubin	1.697 (1.394–2.066)	<0.0001
Tumour number		
Solitary	1	
Multiple	1.284 (1.151–1.432)	<0.0001
Age groups		
Under 51	1	
51–60	0.946 (0.793–1.130)	0.541
61–70	0.943 (0.791–1.123)	0.508
Over 70	1.209 (1.000–1.463)	0.05
Aetiology		
HCV	1	
HBV	1.233 (1.050–1.449)	0.011
Other	1.231 (1.076–1.408)	0.002

Abbreviations: AFP=alphafetoprotein; CI=confidence interval; HCV=hepatitis C virus; HBV=hepatitis B virus.

Theta=0.0185, LR test of theta=0, chibar2(01)=17.83, *P*<0.0001.

**Table 4:** Median survival according to each classification system in the various cohorts

**Cohort**	**Classification system**	***N***	**Median survival in months (95% CI)**	**Harrell's C**	**AIC**
**Figure 1**					
Europe	ALBI grade 1	384	26.18 (22.93, 28.09)	0.5749	11269.51
	ALBI grade 2	731	14.61 (13.39, 15.92)		
	ALBI grade 3	82	9.70 (5.80, 14.01)		
Japan	ALBI grade 1	85	38.91 (27.27, 51.35)	0.566	5816.714
	ALBI grade 2	462	22.43 (19.61, 25.39)		
	ALBI grade 3	106	15.33 (9.34, 20.46)		
Egypt	ALBI grade 1	156	30.00 (20.00,.)	0.5808	4443.527
	ALBI grade 2	690	18.00 (17.00, 20.00)		
	ALBI grade 3	152	13.00 (9.00, 14.00)		
Hong Kong (China)	ALBI grade 1	34	30.20 (16.84, 47.17)	0.5898	995.5034
	ALBI grade 2	98	18.65 (12.60, 25.59)		
	ALBI grade 3	11	6.05 (1.22, 14.18)		
**Figure 2**					
All	ALBI grade 1	659	27.60 (25.53, 29.90)	0.5661	26963.33
	ALBI grade 2	1981	17.76 (16.94, 18.62)		
	ALBI grade 3	351	12.40 (9.61, 14.18)		
All	ALBI grade 1 (within C-P A)	626	27.86 (25.95, 30.00)	0.5478	14901.81
	ALBI grade 2 (within C-P A)	1146	19.05 (17.99, 20.63)		
	ALBI grade 3 (within C-P A)	12	18.09 (6.88,.)		
All	C-P A	1791	21.78 (20.00, 23.65)	0.5586	26548.21
	C-P B	1055	15.20 (14.00, 16.28)		
	C-P C	118	8.26 (5.00, 12.40)		
All	HAP class A	349	32.96 (28.00, 37.57)	0.6121	22273.51
	HAP class B	768	23.49 (20.00, 26.22)		
	HAP class C	955	18.00 (16.81, 19.14)		
	HAP class D	523	11.91 (9.34, 12.00)		
All	Without macrovascular invasion	2567	20.39 (19.51, 22.00)	NA	NA
	With macrovascular invasion	447	8.22 (7.34, 9.87)	NA	NA
Japan	VP0	450	28.19 (25.82, 31.11)	NA	NA
	VP1	60	18.62 (13.22, 21.78)	NA	NA
	VP2/3	110	9.54 (7.43, 11.58)	NA	NA
	VP4	16	4.14 (2.37, 8.91)	NA	NA

Abbreviations: AIC=Akaike information criterion; CI=confidence interval; C-P=Child-Pugh; HCV=hepatitis C virus; HBV=hepatitis B virus.

## Supplementary Material

Supplementary Table 3

Supplementary Figure 1A

Supplementary Figure 1B

Supplementary Figure 1C

Supplementary Figure 1D

Supplementary Figure 2A

Supplementary Figure 2B

Supplementary Figure 2C

Supplementary Figure 2D

Supplementary Figure 3A

Supplementary Figure 3B

Supplementary Figure 3C

Supplementary Figure 3D

Supplementary Figure 4A

Supplementary Figure 4B

Supplementary Figure 4C

Supplementary Figure 4D
