# Supplementary material for: Transarterial chemo-embolisation of hepatocellular carcinoma: impact of liver function and vascular invasion
Source: Br J Cancer. 2017 Jan 26;116(4):448–54. doi: 10.1038/bjc.2016.423 (PMC5318968; doi:10.1038/bjc.2016.423)
Supplement: Supplementary Figures [file bjc2016423x1.docx]

***Supplementary figure legends***

**Supplementary figure 1:** Kaplan-Meier curves depicting survival according to ALBI grade (within C-P A) in patients undergoing TACE from (a) Europe, (b) Japan, (c) Egypt and (d) Hong Kong, China.

**Supplementary figure 2:** Kaplan-Meier curves depicting survival according to C-P grade in patients undergoing TACE from (a) Europe, (b) Japan, (c) Egypt and (d) Hong Kong, China.

**Supplementary figure 3:** Kaplan-Meier curves depicting survival according to HAP class in patients undergoing TACE from (a) Europe, (b) Japan, (c) Egypt and (d) Hong Kong, China.

**Supplementary figure 4:** Kaplan-Meier curves depicting survival according to macrovascular invasion in patients undergoing TACE from (a) Europe, (b) Japan, (c) Egypt and (d) Hong Kong, China.
