# Supplementary figures and images for: Transarterial chemo-embolisation of hepatocellular carcinoma: impact of liver function and vascular invasion
Source: Br J Cancer. 2017 Jan 26;116(4):448–54. doi: 10.1038/bjc.2016.423 (PMC5318968; doi:10.1038/bjc.2016.423)

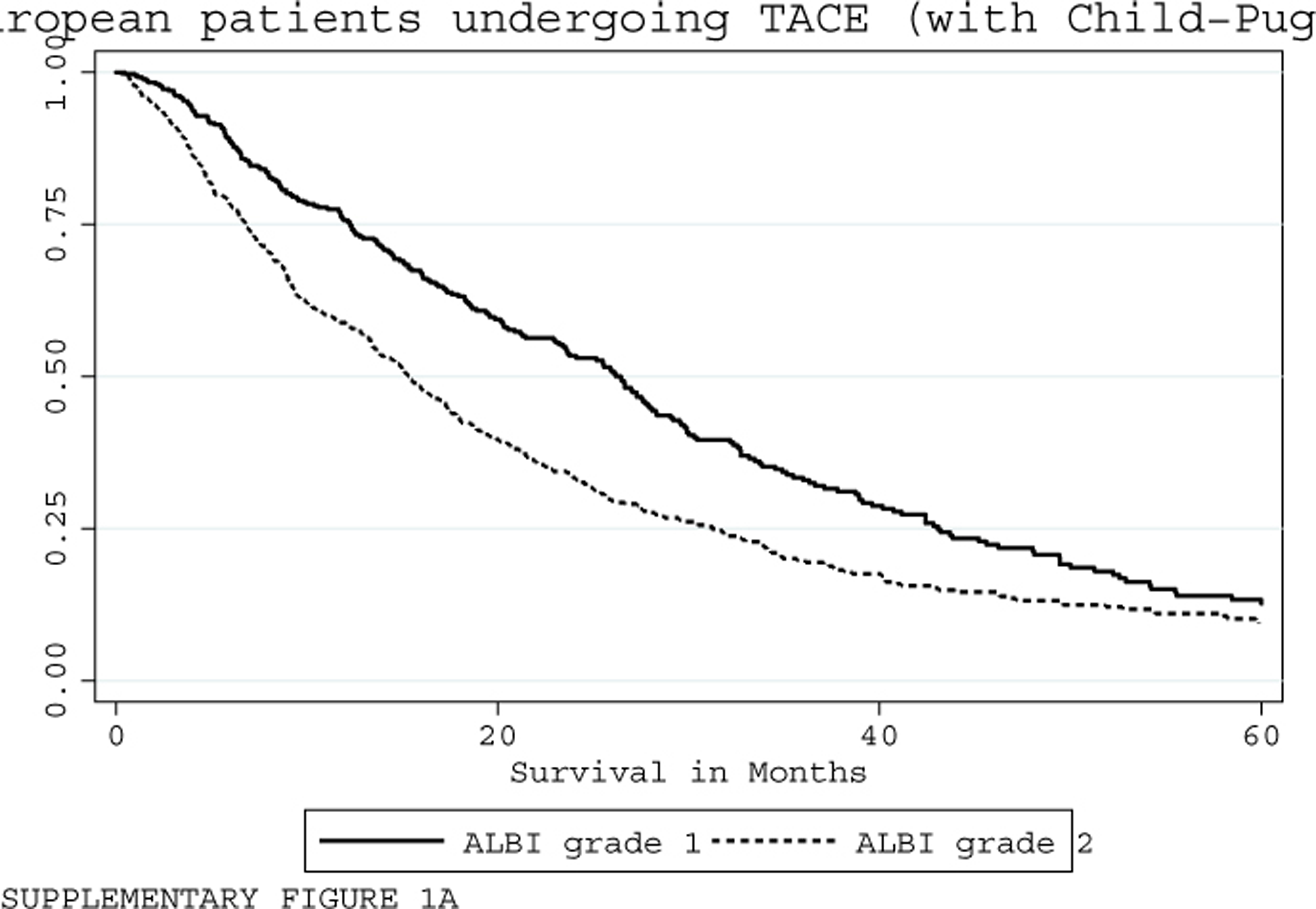

Supplement: Supplementary Figure 1A [file bjc2016423x5.tif]

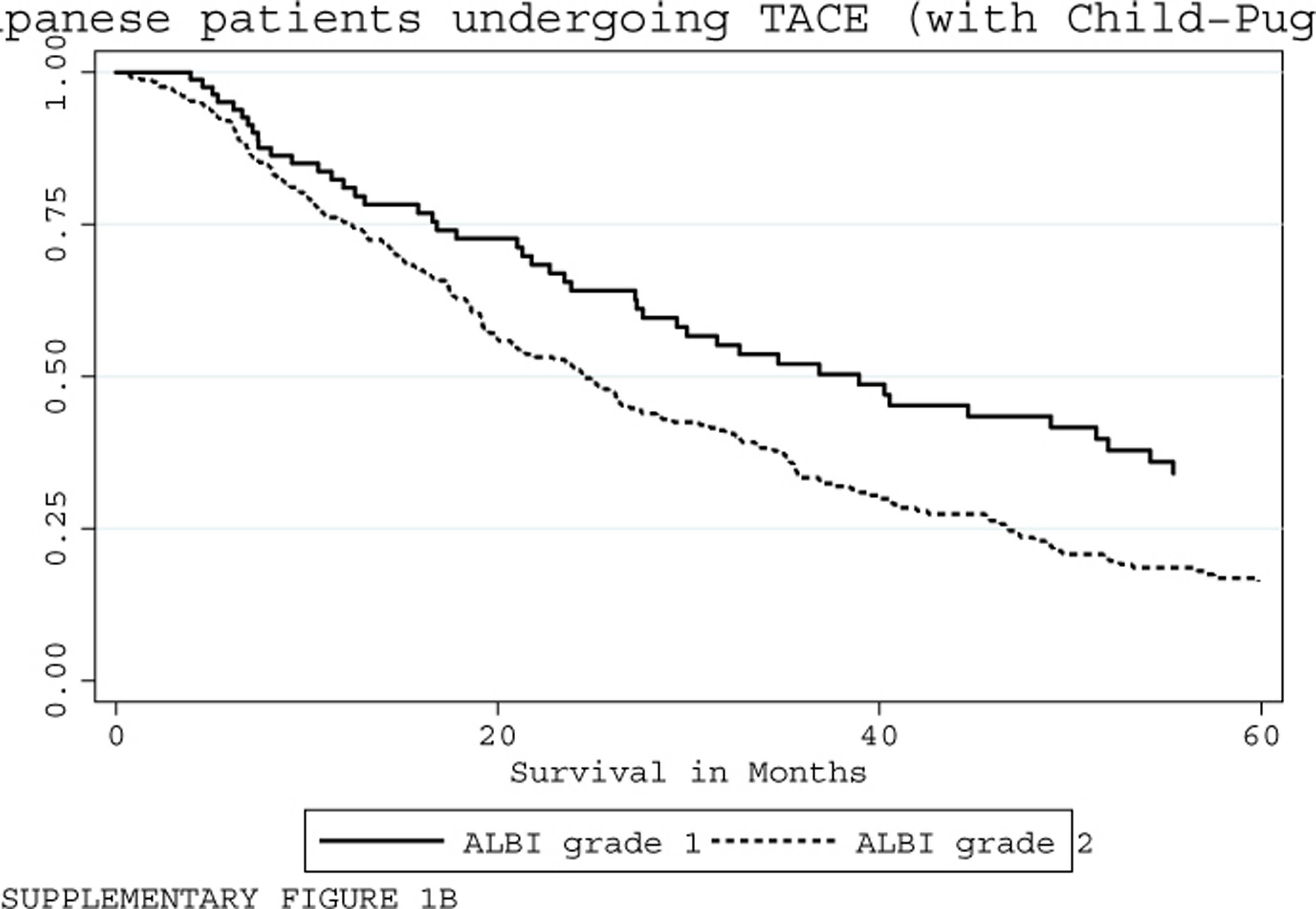

Supplement: Supplementary Figure 1B [file bjc2016423x6.tif]

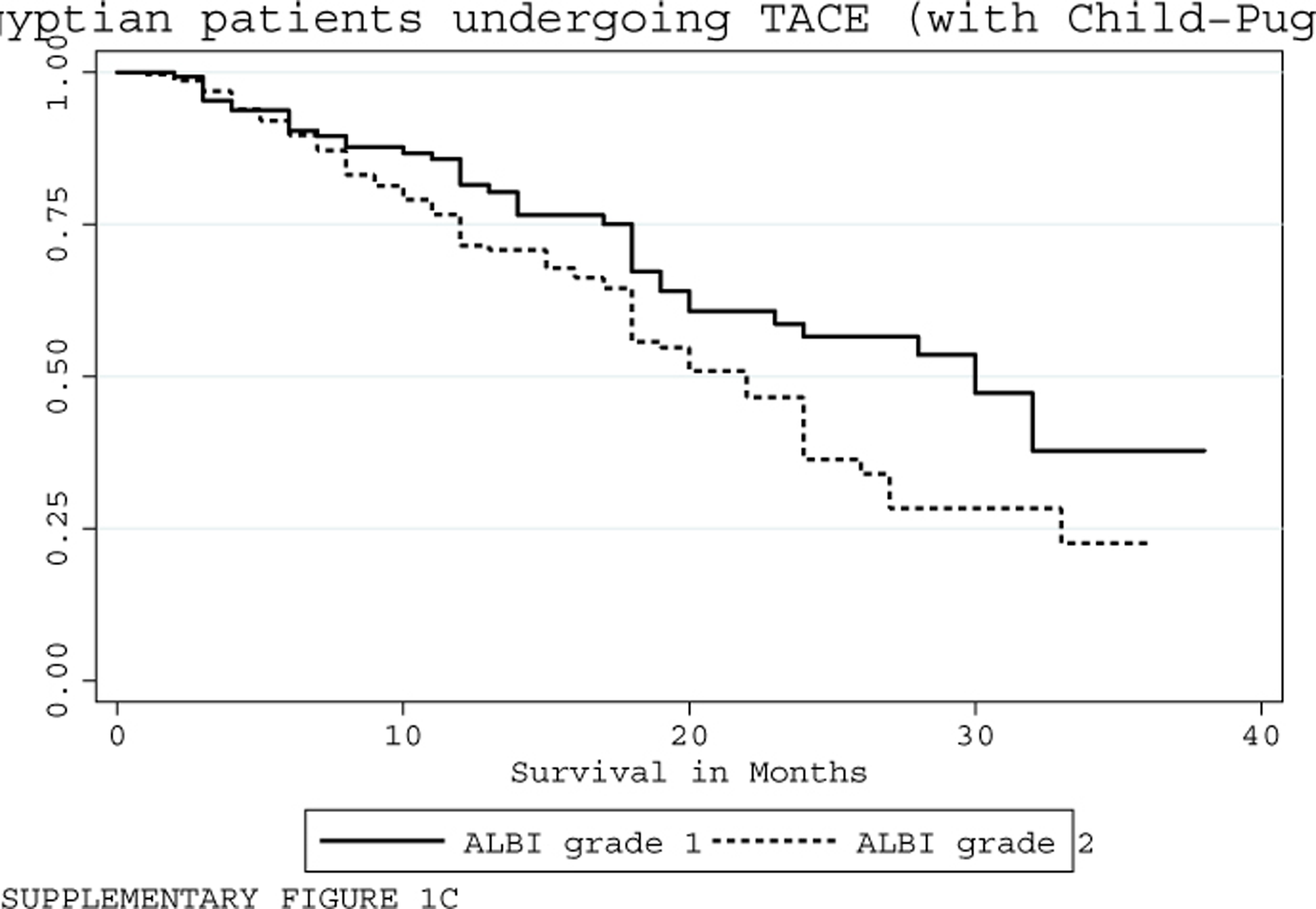

Supplement: Supplementary Figure 1C [file bjc2016423x7.tif]

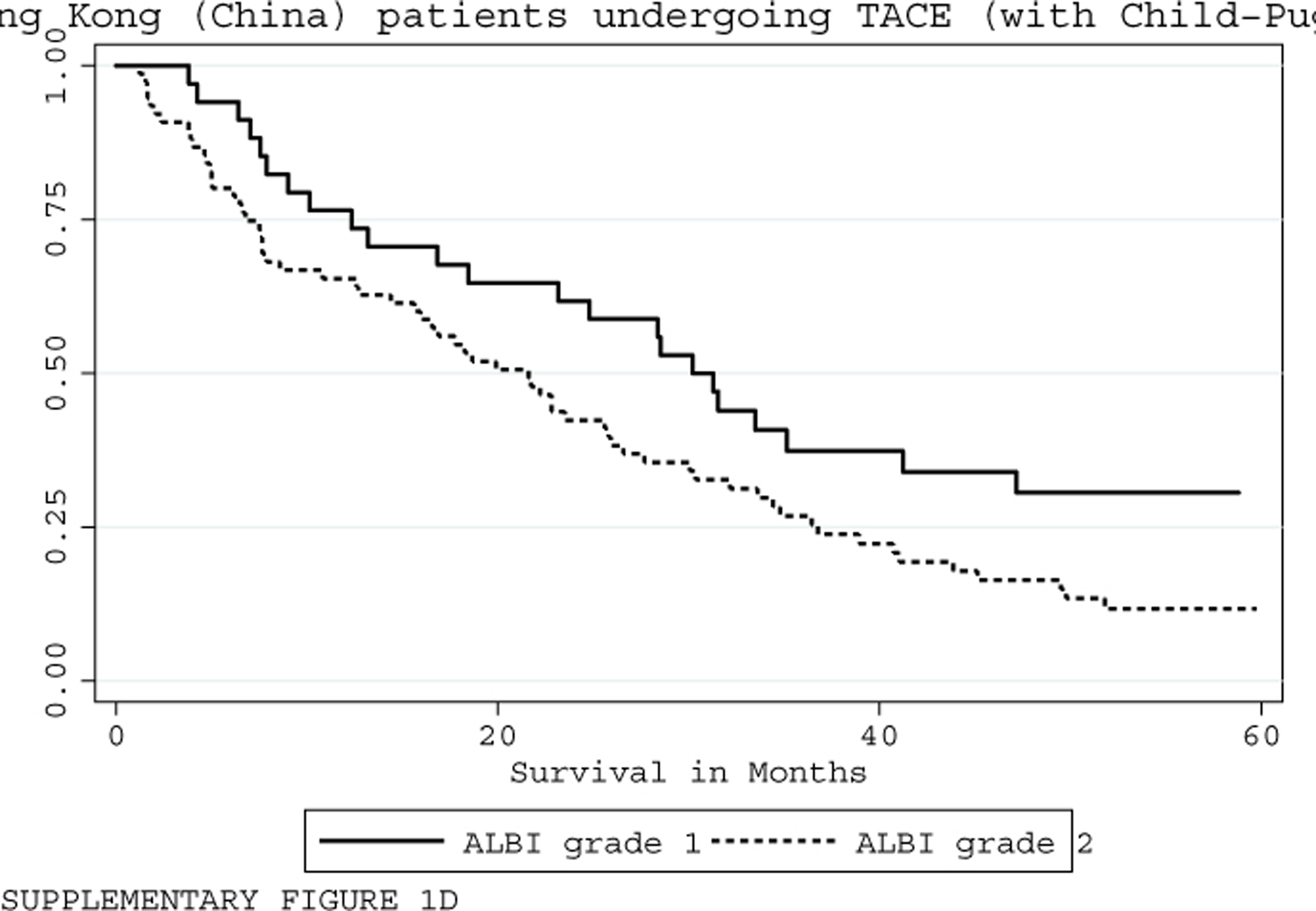

Supplement: Supplementary Figure 1D [file bjc2016423x8.tif]

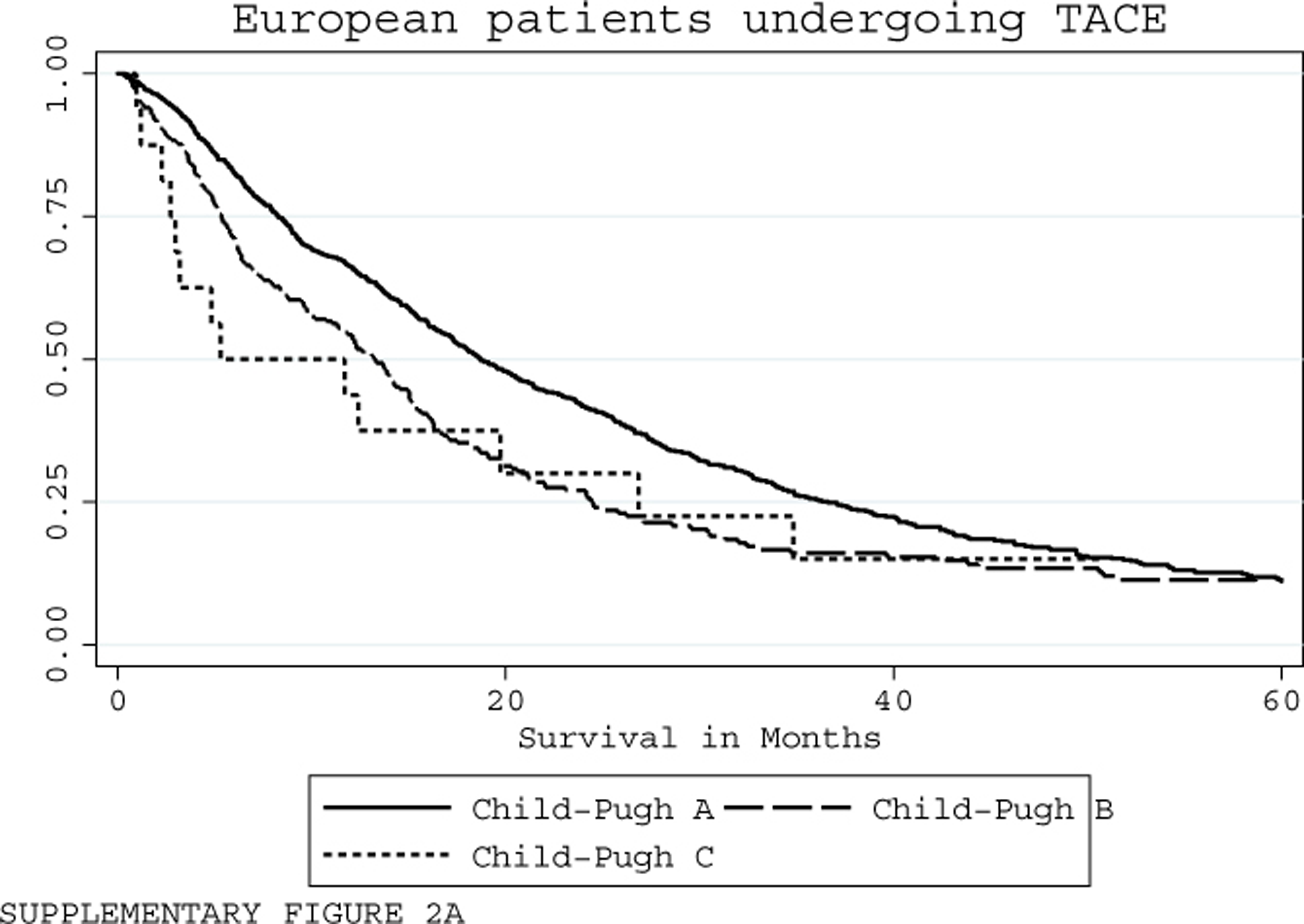

Supplement: Supplementary Figure 2A [file bjc2016423x9.tif]

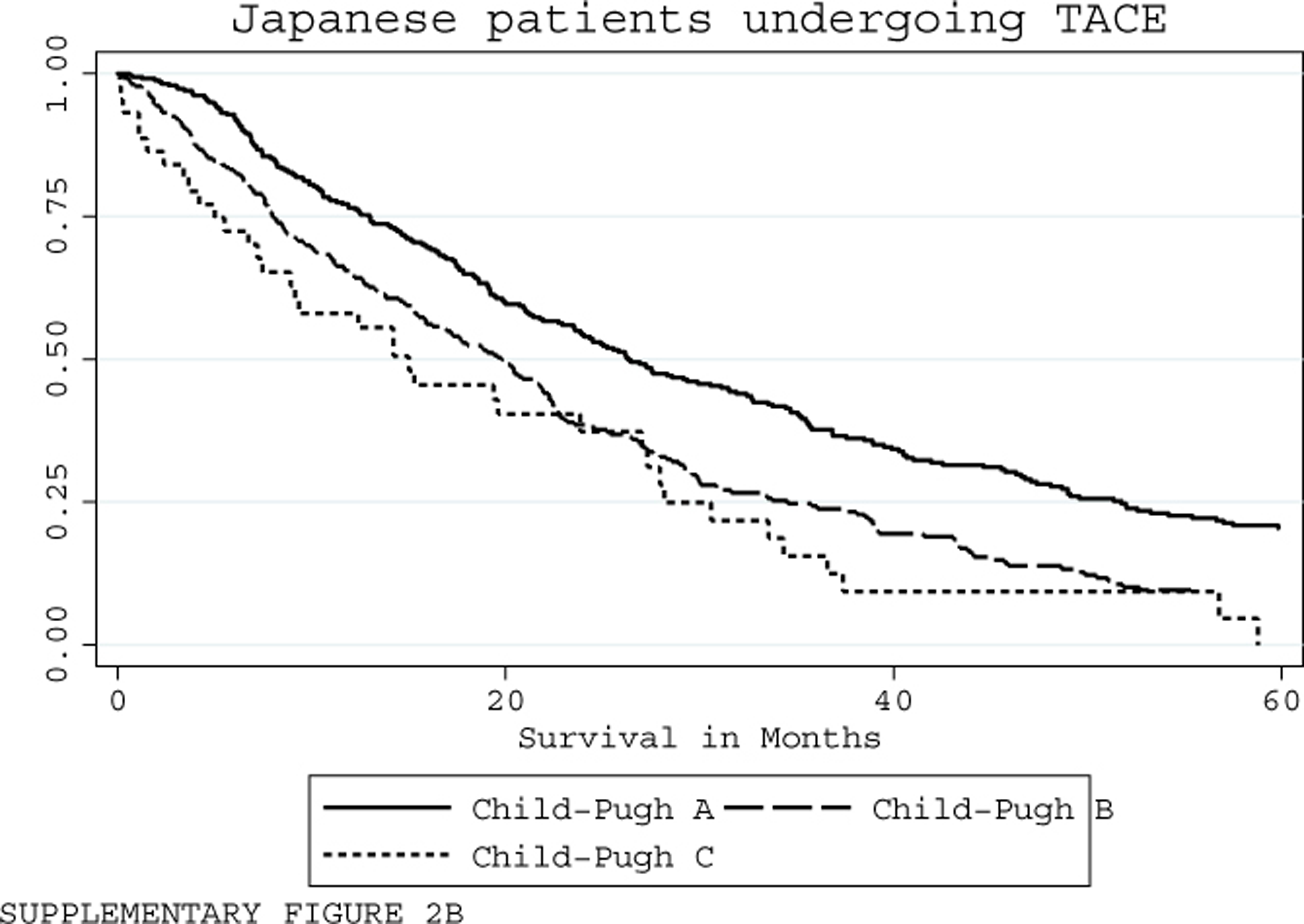

Supplement: Supplementary Figure 2B [file bjc2016423x10.tif]

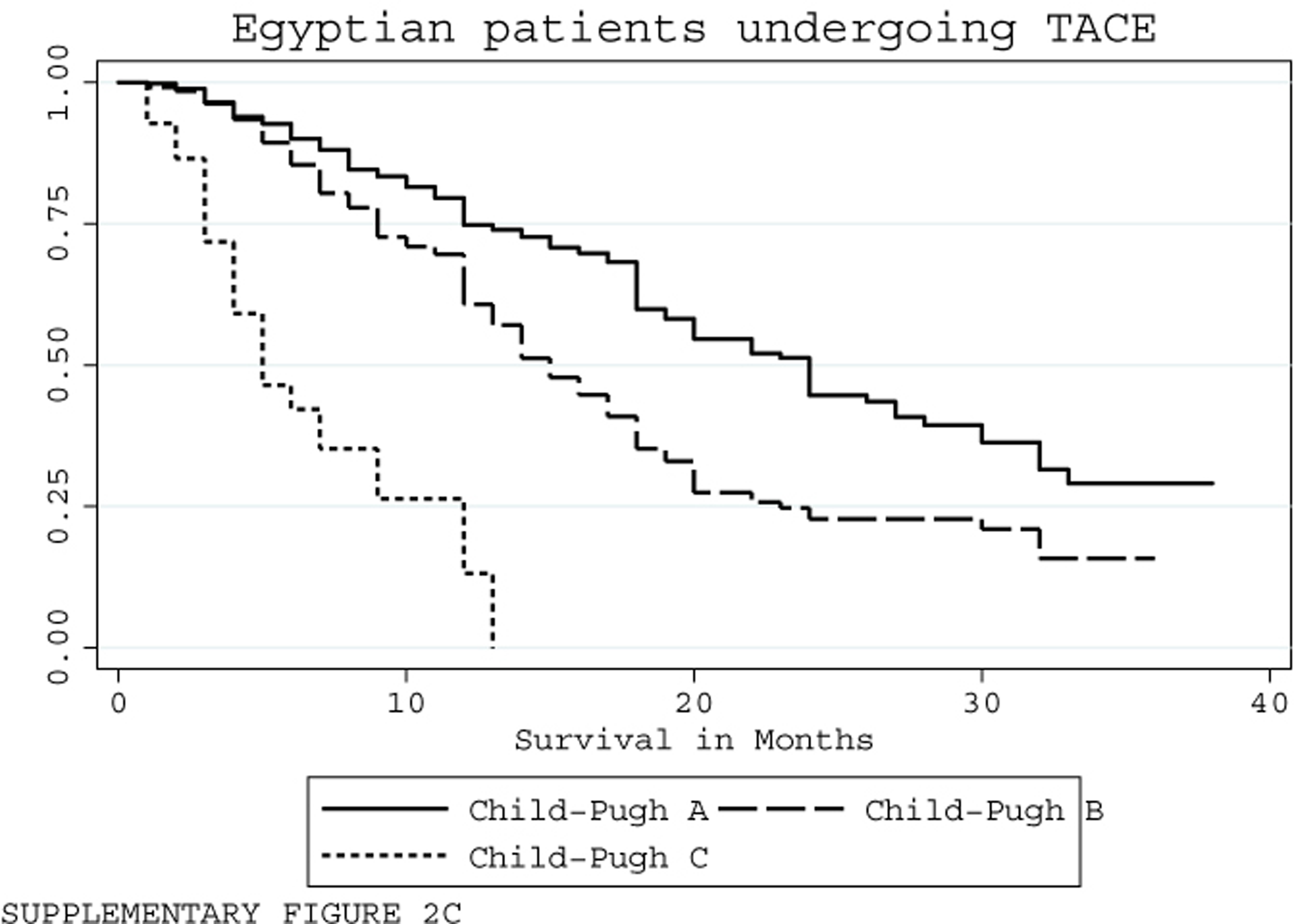

Supplement: Supplementary Figure 2C [file bjc2016423x11.tif]

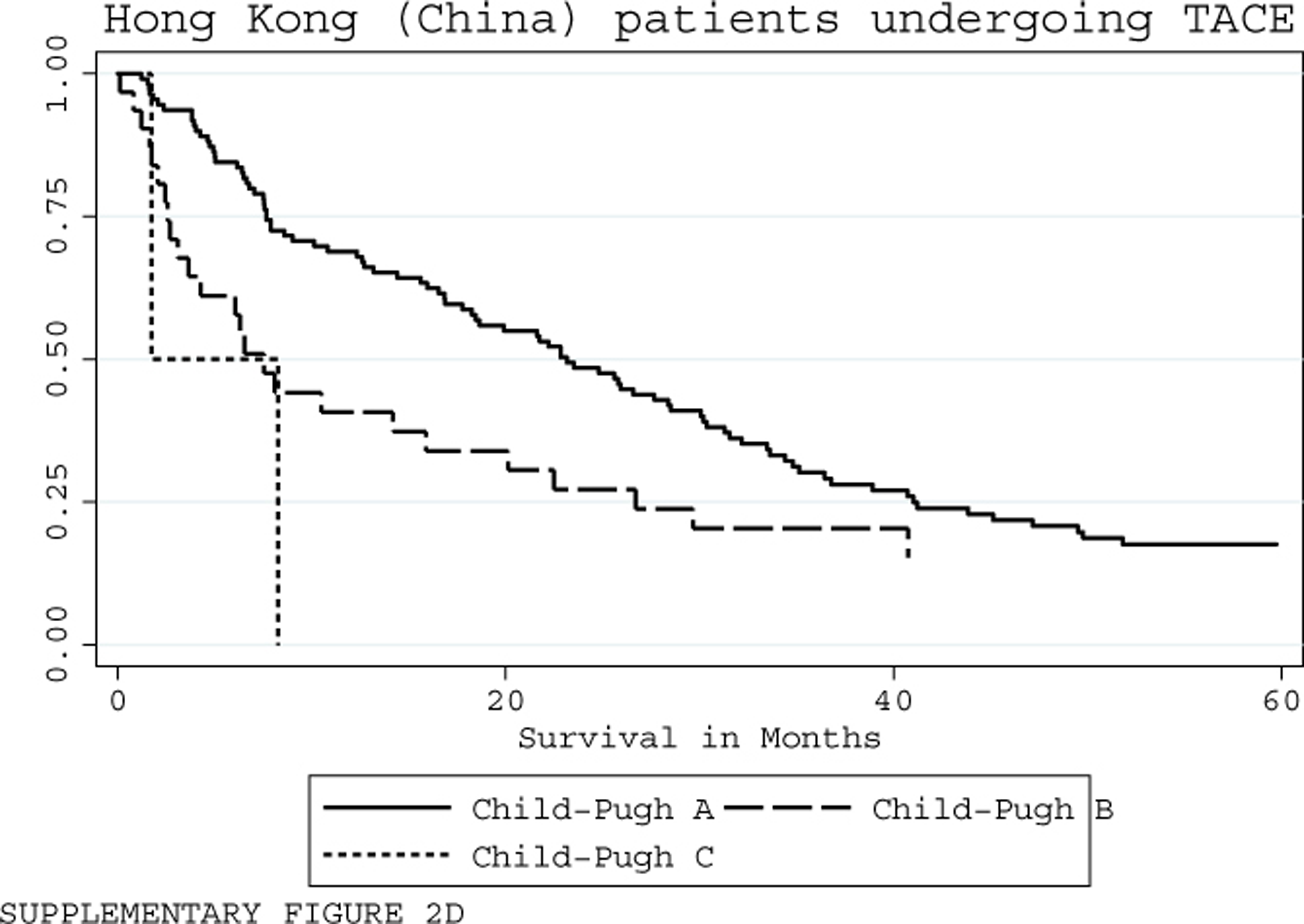

Supplement: Supplementary Figure 2D [file bjc2016423x12.tif]

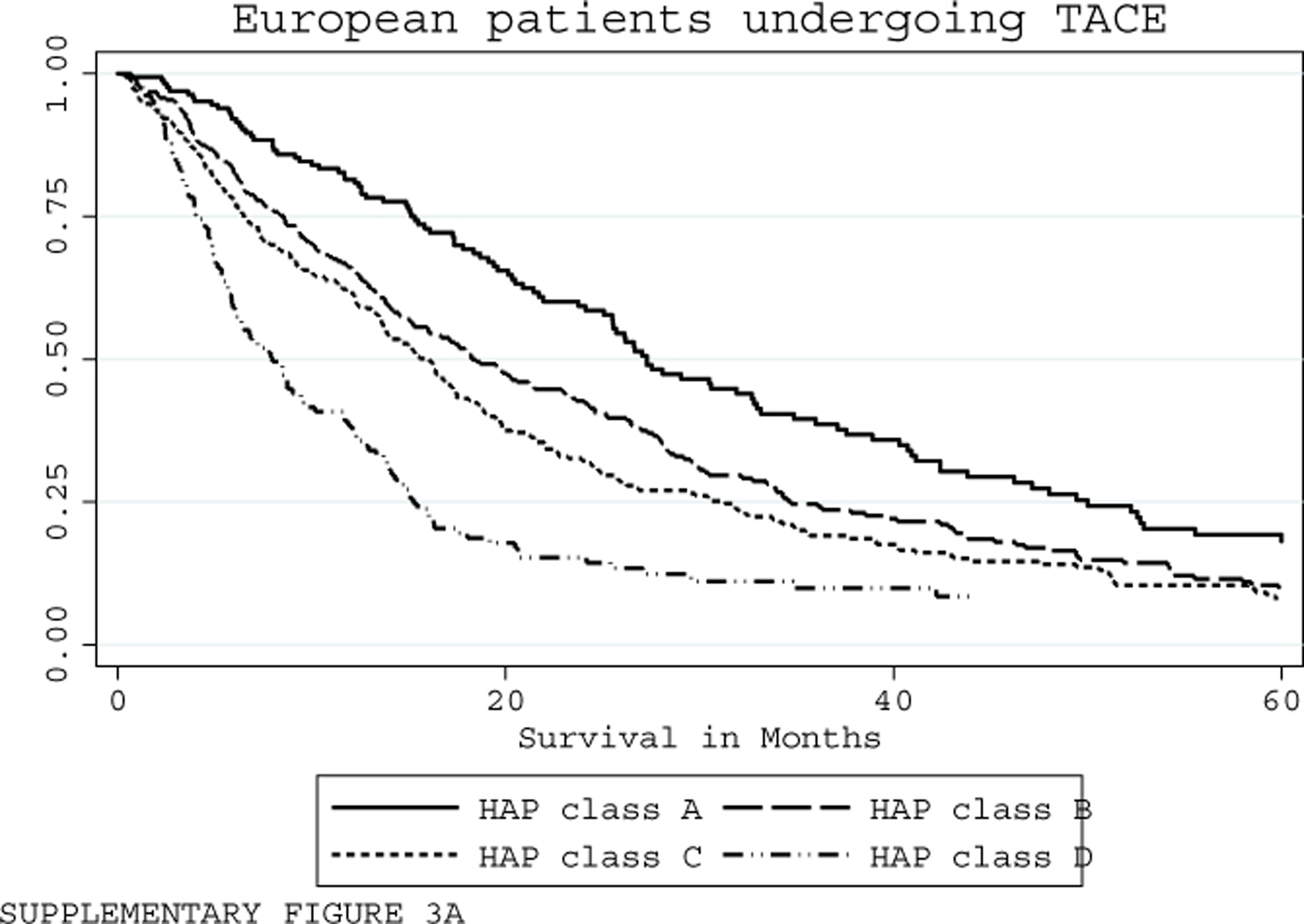

Supplement: Supplementary Figure 3A [file bjc2016423x13.tif]

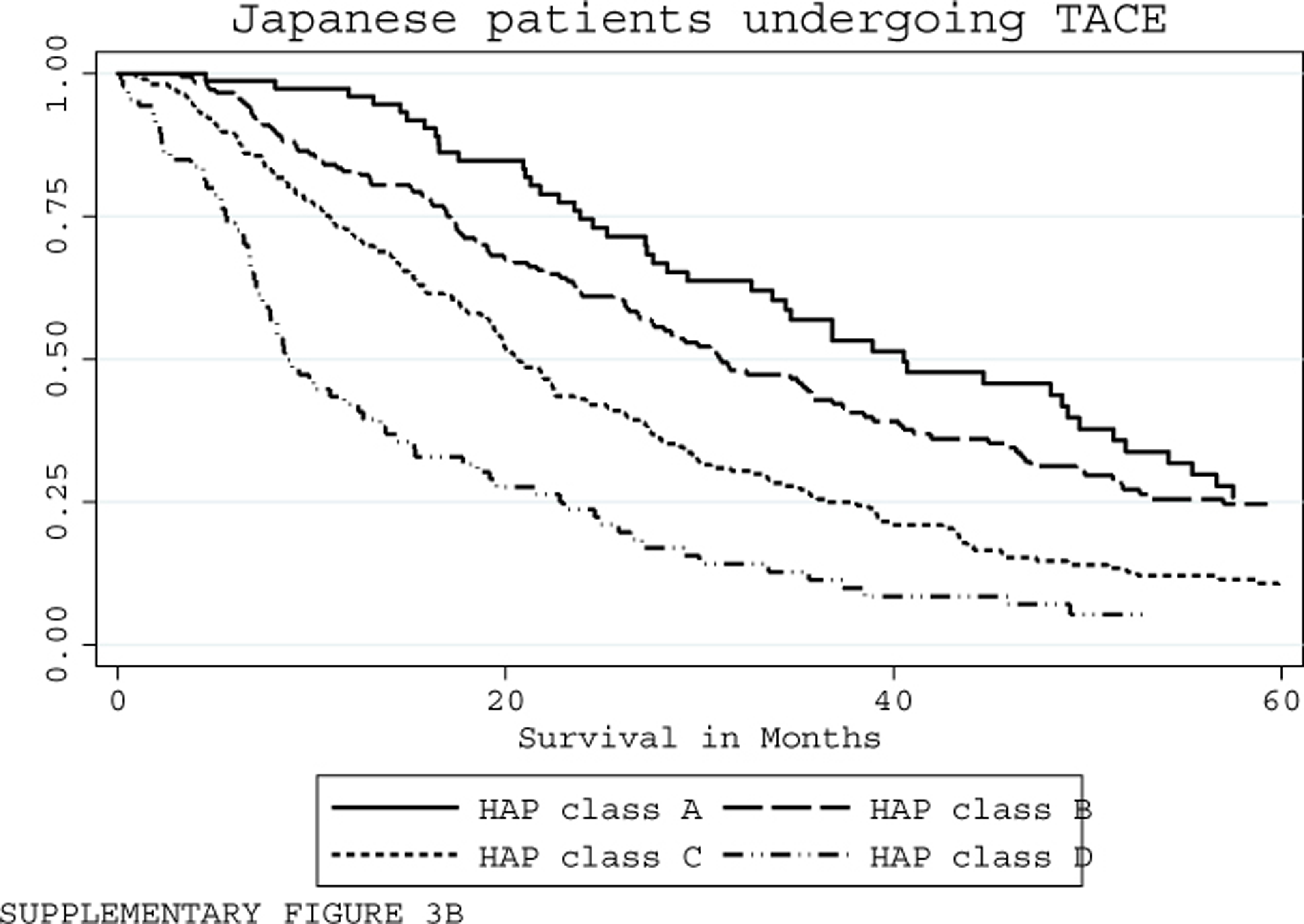

Supplement: Supplementary Figure 3B [file bjc2016423x14.tif]

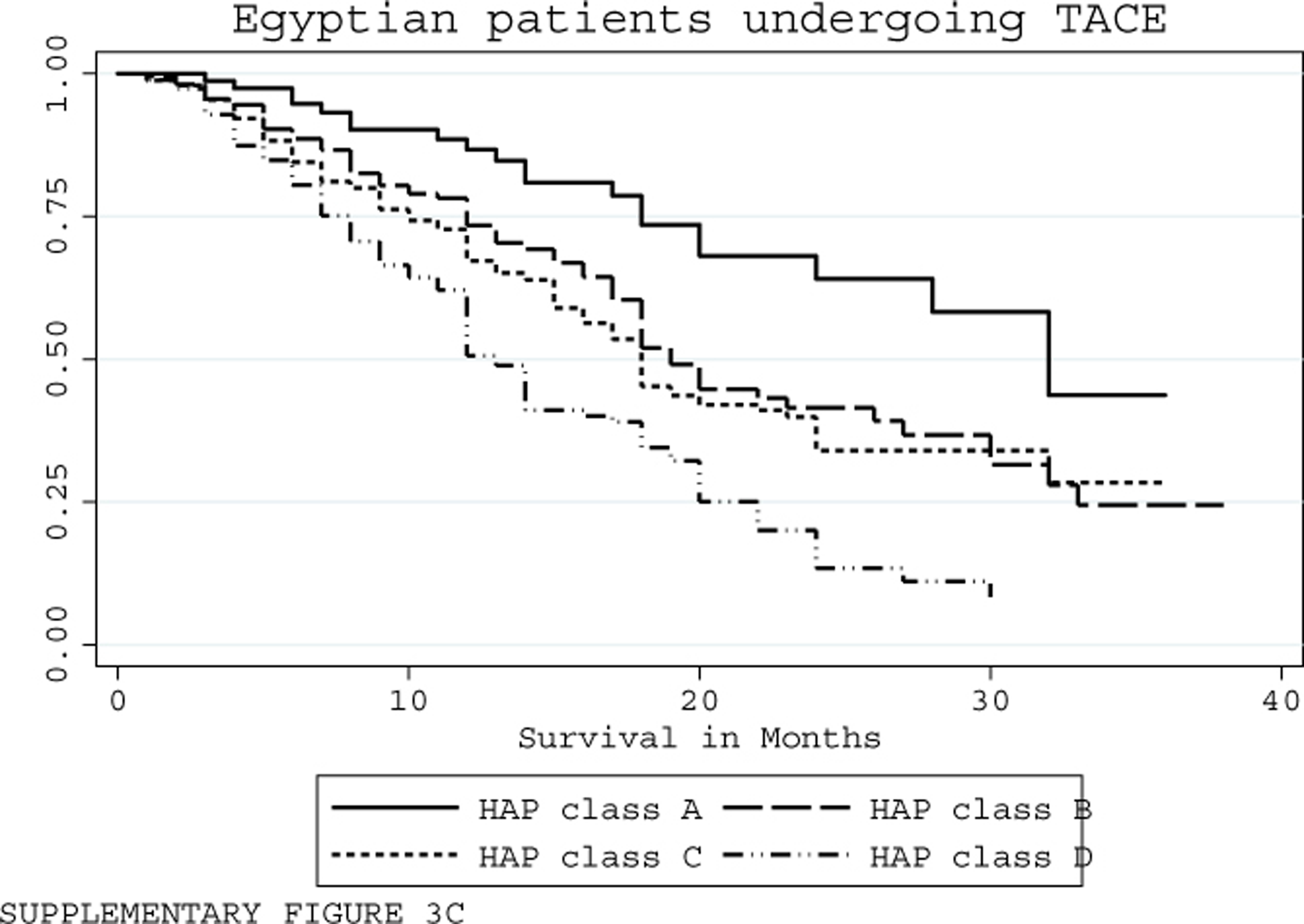

Supplement: Supplementary Figure 3C [file bjc2016423x15.tif]

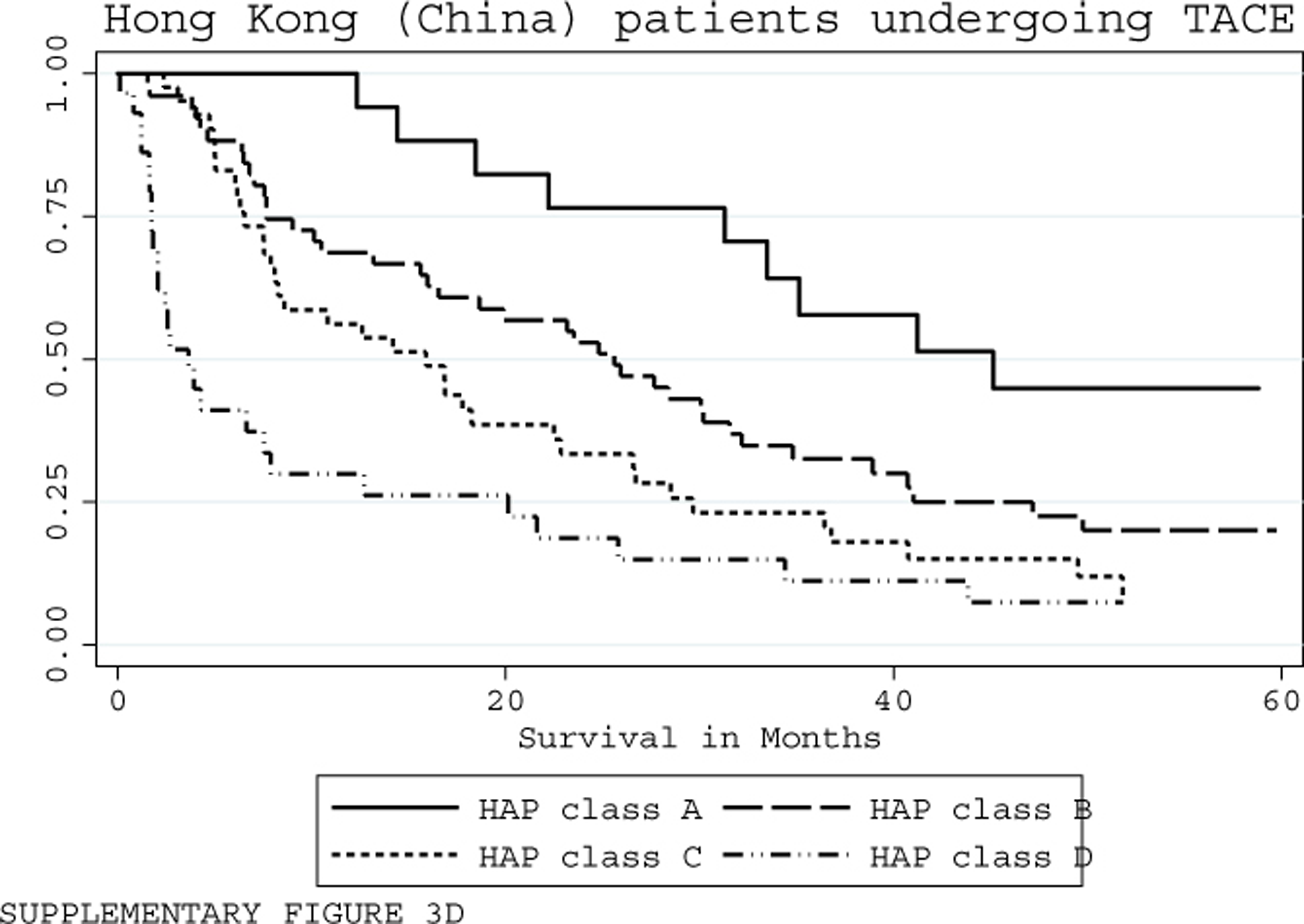

Supplement: Supplementary Figure 3D [file bjc2016423x16.tif]

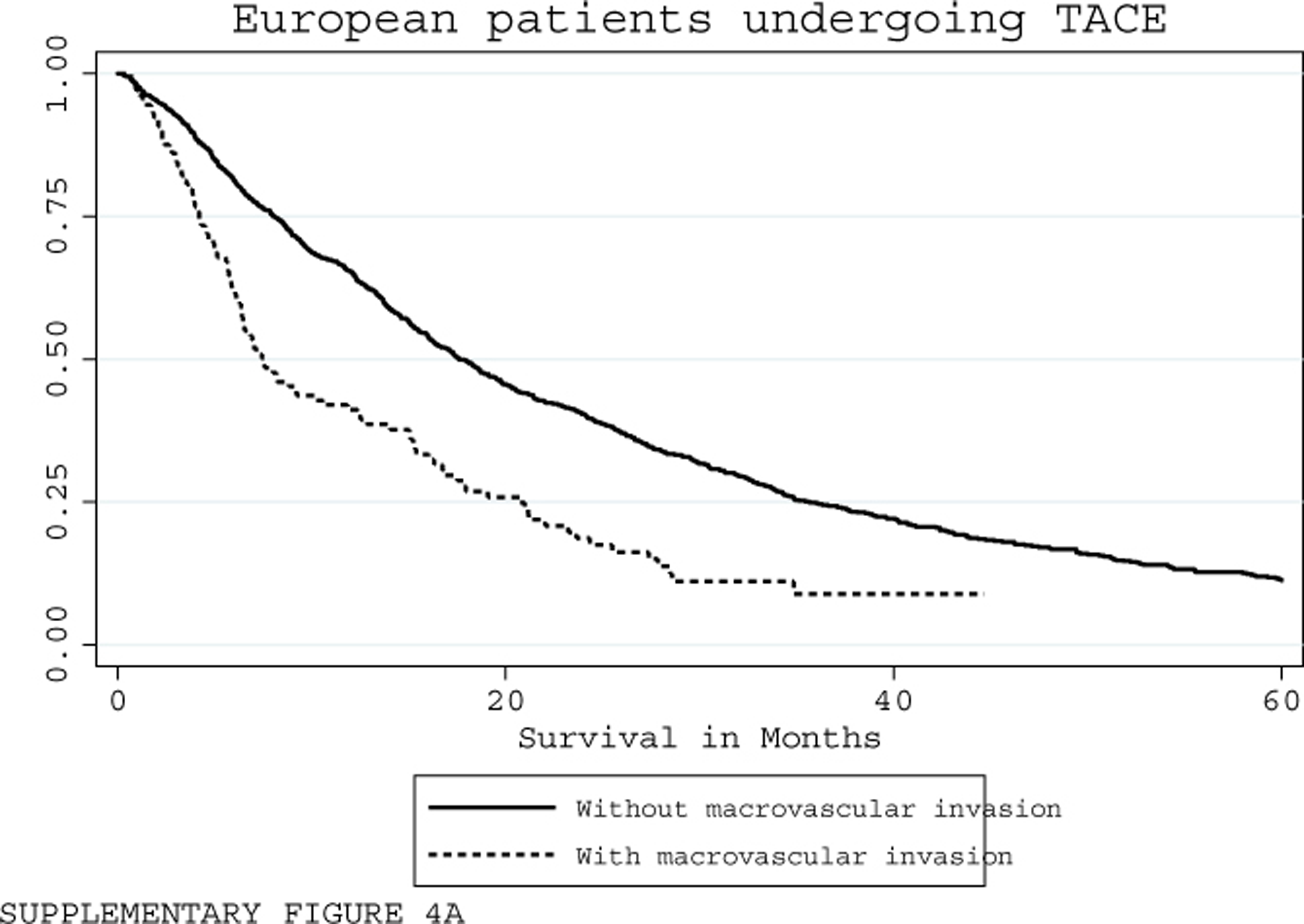

Supplement: Supplementary Figure 4A [file bjc2016423x17.tif]

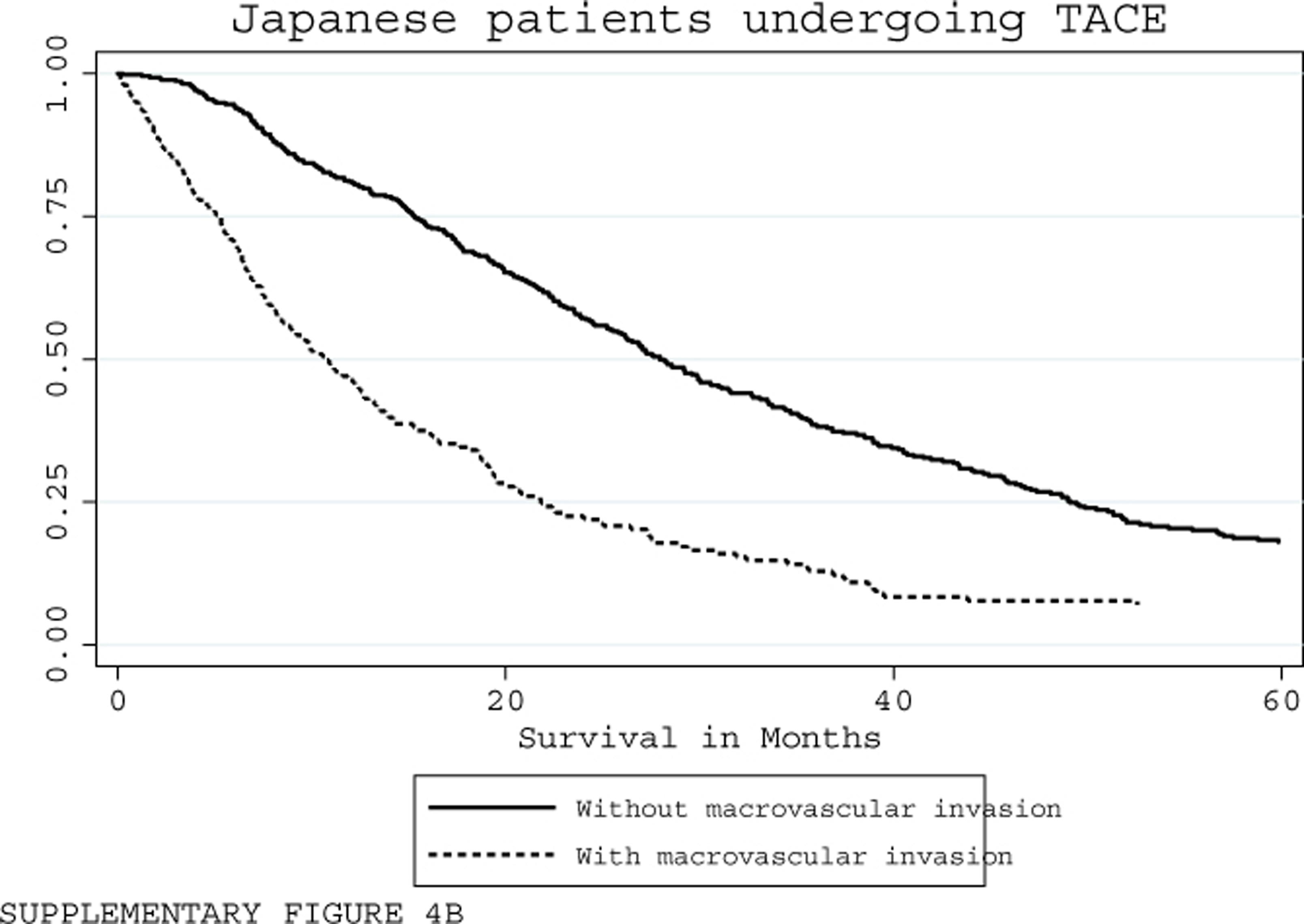

Supplement: Supplementary Figure 4B [file bjc2016423x18.tif]

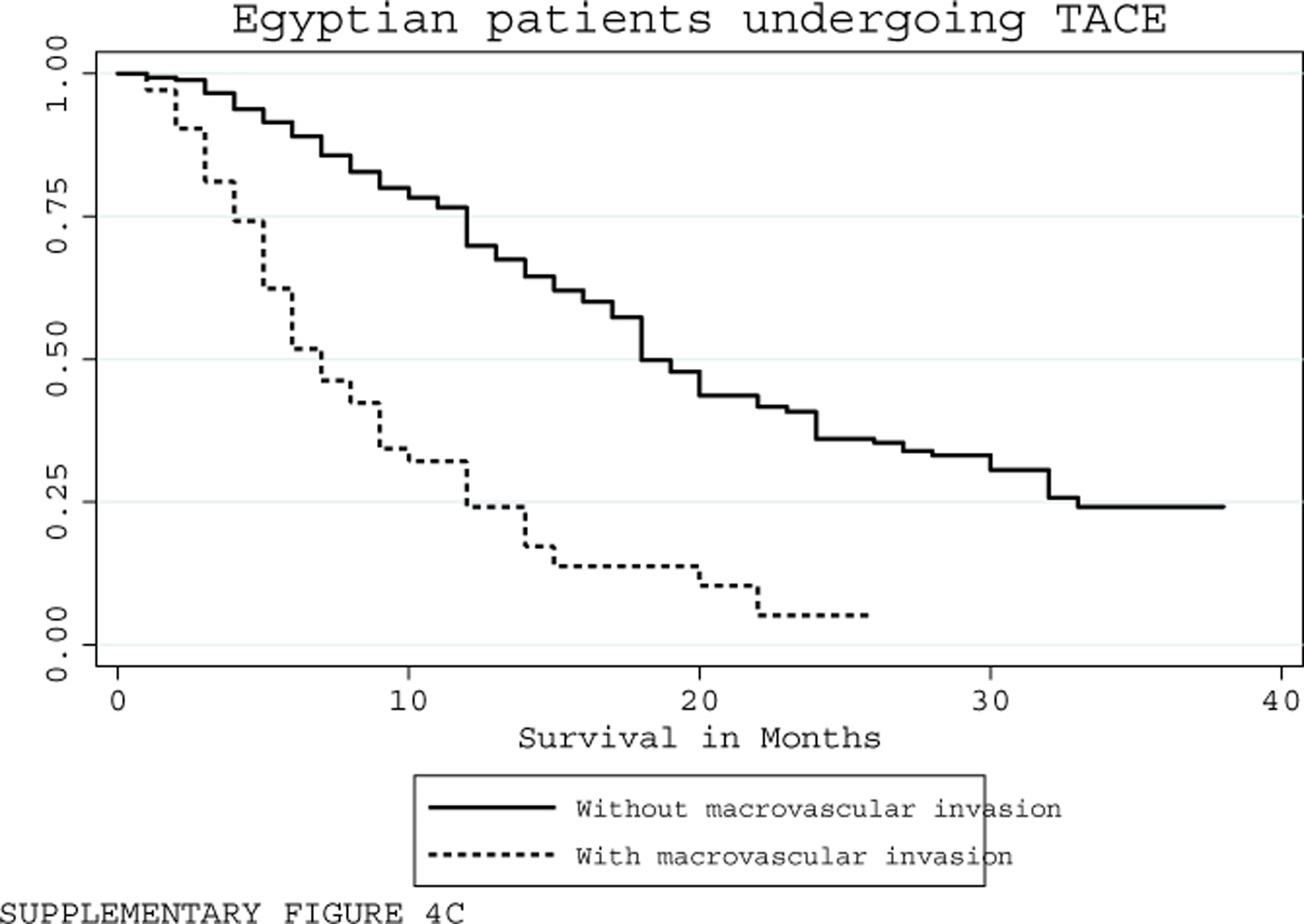

Supplement: Supplementary Figure 4C [file bjc2016423x19.tif]

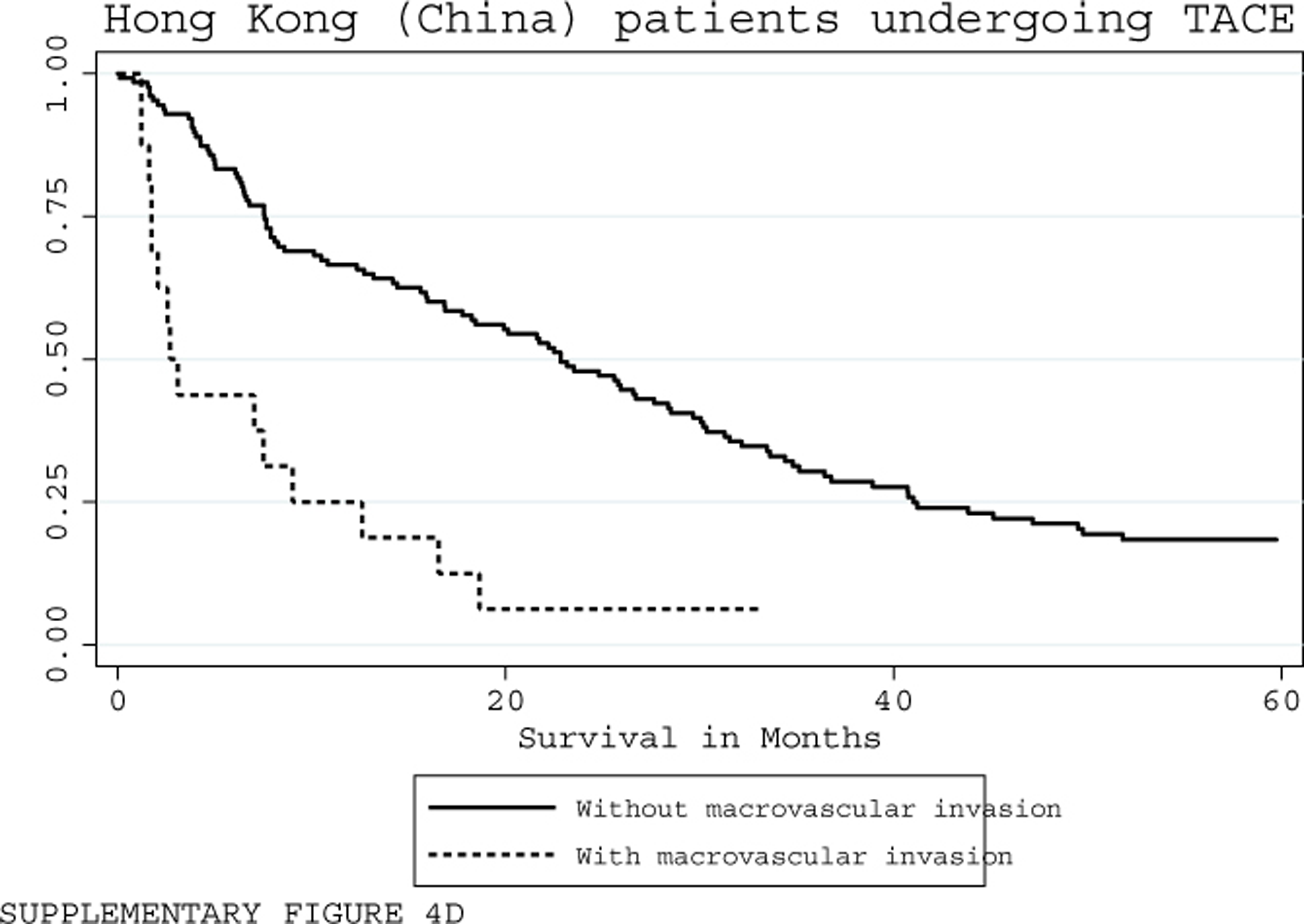

Supplement: Supplementary Figure 4D [file bjc2016423x20.tif]
